# A Survey of Computational Intelligence Techniques in Protein Function Prediction

**DOI:** 10.1155/2014/845479

**Published:** 2014-12-11

**Authors:** Arvind Kumar Tiwari, Rajeev Srivastava

**Affiliations:** Department of Computer Science & Engineering, Indian Institute of Technology (BHU), Varanasi 221005, India

## Abstract

During the past, there was a massive growth of knowledge of unknown proteins with the advancement of high throughput microarray technologies. Protein function prediction is the most challenging problem in bioinformatics. In the past, the homology based approaches were used to predict the protein function, but they failed when a new protein was different from the previous one. Therefore, to alleviate the problems associated with homology based traditional approaches, numerous computational intelligence techniques have been proposed in the recent past. This paper presents a state-of-the-art comprehensive review of various computational intelligence techniques for protein function predictions using sequence, structure, protein-protein interaction network, and gene expression data used in wide areas of applications such as prediction of DNA and RNA binding sites, subcellular localization, enzyme functions, signal peptides, catalytic residues, nuclear/G-protein coupled receptors, membrane proteins, and pathway analysis from gene expression datasets. This paper also summarizes the result obtained by many researchers to solve these problems by using computational intelligence techniques with appropriate datasets to improve the prediction performance. The summary shows that ensemble classifiers and integration of multiple heterogeneous data are useful for protein function prediction.

## 1. Introduction

Protein function prediction is a very important and challenging task in bioinformatics. Protein is the most important molecule in our life. It is responsible for structuring the organs, catalysis of biochemical reaction for metabolism, and maintenance of cellular components. The knowledge of the functionality of a protein is very important to develop new approaches in any biological process. The experiment based protein function prediction required a huge experimental and human effort to analyze a single gene or protein. So to remove this drawback a number of very high throughput experimental procedures have been invented to investigate the methods that are used in function prediction. These procedures have generated a variety of data, such as protein sequences, protein structures, protein interaction network, and gene expression data used in function prediction. There are many databases to maintain these data, such as SWISS-PROT [1], DIP [2], NCBI [3], STRING [4], and PDB [5].

The homology based methods used the structure of a protein and it identifies the protein with most similar structure using structural alignment techniques. The global and local sequence alignment techniques have been proposed in papers [6–8] and the sequence motifs have been proposed in papers [9, 10] for protein function prediction. The BLAST in paper [6] and FASTA in paper [11] have been proposed for the comparison of amino acid sequences. The position specific score matrix (PSSM) was used in paper [7] to search the protein databases which provide high sensitivity for detecting remote homologs. In paper [12] the authors have observed that the proteins that diverged from a common ancestral gene may have the same function but no detectable sequence similarity. Therefore the sequence similarity based approaches may not always be adequate for protein function prediction.

The protein structures are more conserved than sequences so when a sequence based function prediction cannot be achieved with high accuracy then three-dimensional structures of proteins are used for protein function prediction. The structure of a protein determines several functional features such as cellular location, overall fold, active site residues and their conformation in enzymes, and interactions with ligands and other protein. In paper [13] the authors have used the fold information depending on global and local structural alignment algorithms. The global and local conformational similarities between proteins indicate functional similarities and are useful for inferring functions of proteins. The authors of paper [14] have developed a molecular binding site prediction method which integrates sequence conservation estimates with structure based methods to identify protein surface cavities, ligand binding packets, individual ligand binding residues, catalytic sites, and drug binding pockets. The structural properties based protein function prediction is useful for single static structure and is not useful in dynamic structure, but structural dynamics can enhance the function prediction, so in paper [15] the authors have used molecular dynamics simulation with structure based function prediction algorithm to find the binding sites for protein function prediction. But the availability of high-resolution structural data of the proteins or their homologues is the major limitation of this type of protein function prediction methods.

Sequence and structure based methods used homology relationships among proteins for protein function prediction. When sequence based homology failed, then structure based homology is used to predict protein function. But these methods have problems in protein function prediction due to availability of adequate data of homologous proteins and homologous proteins may have a different function. So these methods fail when homology relationships cannot be established for target proteins [16]. Protein function prediction based on the structure has been restricted in scope because of the availability of a limited number of structures and folds in the databases. Protein function prediction from sequence is a great challenge for the protein that has low or no sequence similarity to proteins of known function. So computational intelligence techniques have been found useful in protein function prediction by using sequence derived properties independent of sequence similarity that have a great potential for low and nonhomologous protein [17].

The advancement of high throughput technologies has produced a large amount of high throughput data such as protein-protein interaction and gene expression data that are also useful for protein functions prediction. Gene expression measurements provide the genes which are active under certain condition that produces a protein to perform a given function under such conditions. So it is expected that coexpressed genes perform similar cellular functions. Various computational intelligence techniques are used to annotate unknown genes that coexpress with known genes. Proteins perform a specific function by interacting with another protein. So protein-protein interaction network provide valuable data that is useful in protein function prediction. Pathway consists of genes that chemically act together for specific cellular or physiological functions so pathway analysis of gene expression data is also useful for gene function prediction.

There are various online servers which are also available to predict the various protein functions. The BPBind (http://lcg.rit.albany.edu/dp-bind/) [18] and BindN (http://bioinfo.ggc.org/bindn/) [19] are the web servers to predict binding sites using sequence derived properties of protein sequence. The SVMPort (http://jing.cz3.nus.edu.sg/cgi-bin/svmprot.cgi) [20] is the web server to predict protein function by using sequence and structure derived properties of protein sequence. The GPCRsclass (http://www.imtech.res.in/raghava/gpcrsclass/) [21] is the SVM based web server to predict the G-protein coupled receptors and their subfamilies by using amino acid and dipeptide composition of protein sequences. MemType-2L (http://www.csbio.sjtu.edu.cn/bioinf/MemType/) [22] is the ensemble classifier based web server to predict the membrane proteins by using pseudoamino acid composition.

## 2. An Overview of Computational Intelligence Techniques in Protein Function Prediction by Using Sequence and Structure

This section presents a state-of-the-art comprehensive review of various computational intelligence techniques used in wide areas of applications such as prediction of DNA and RNA binding sites, subcellular localization, enzyme functions, signal peptides, catalytic residue, nuclear/G-protein coupled receptors, and membrane proteins by using sequence and structure; it also presents the summary of the result obtained by many researchers to solve these problems by using computational intelligence techniques with appropriate datasets to improve the prediction performance.

### 2.1. Computational Intelligence Techniques in Prediction of Binding Sites

The interaction of protein-DNA plays the most important role in cellular function. The predictions of DNA-binding sites in proteins are very important for understanding the molecular mechanisms of protein-DNA interaction. So it is necessary to design a robust and efficient computational intelligence techniques based method to predict the DNA binding sites. So for achieving this objective various computational intelligence techniques have been proposed in literatures. Some of the prominent computational intelligence techniques reported in literature for the application under consideration include artificial neural network (ANN), support vector machine (SVM), Naïve Bayes, and random forest based ensemble classifiers based methods. This section of the paper presents an analysis of various research papers in literature and examines the efficacy of each of these methods for the predictions of DNA and RNA-binding sites in proteins which are as follows.

An artificial neural network (ANN) based method has been proposed in papers [23, 24] to predict the DNA binding sites by using information on the amino acid sequence composition, solvent accessibility and secondary structure in paper [23], and position specific scoring matrices (PSSM) in paper [24]. The authors observed that the ANN based method had not obtained the desired level of performance in these works.

The support vector machine (SVM) based methods to predict the DNA binding sites have been proposed in papers [18, 25–29] by using the different sequential and structural features. In paper [25] surface and overall composition, overall charge, and positive potential patches on the protein surface have been used. In paper [26] amino acid sequence, PSSM, and low-resolution structural information have been used. The authors of this paper compared their result by using only sequence, sequence and structure, PSSM, and PSSM with structure. From the comparative analysis it was observed by the authors that the highest prediction accuracy is achieved by using a combination of evolutionary conservation (PSSM) and low-resolution structural information. In paper [18], amino acid sequence and PSSM have been used. In paper [27], amino acid sequence, pseudoamino acid composition, autocross-covariance transforms, and dipeptide composition have been used. In paper [28], normalized PSSM score, normalized solvent accessible surface area, and protein backbone structure have been used. In paper [29], PSSM, amino acid composition, hydrophobicity, polarity, polarizability, secondary structure, solvent accessibility, normalized Vander Waals volume, and binding and nonbinding propensity have been used. In paper [30] the authors have proposed a combination of SVM and ANN based method to predict the DNA binding sites with PSSM and structural features such as secondary structure, solvent accessibility, and globularity. They then compared their results by using only sequence, PSSM, PSSM and sequence, protein-protein interaction data, and PSSM with structural features. It was observed by the authors of the paper that structural information is necessary for improving the prediction performance of DNA binding sites.

The random forest based method to predict the DNA binding residues has been proposed in papers [31–33] by using the different sequential and structural features. In paper [31], mean and standard deviation of amino acid sequence features, side chain pKa value, hydrophobicity index, molecular mass, and PSSM have been used. Then, the result was compared by the authors by using the mean and standard deviation side chain pKa value, hydrophobicity index, and molecular mass with and without PSSM. It was observed by the authors of the paper that result was improved by using the PSSM. In paper [32], PSSM, secondary structure information, orthogonal binary vector information, two physical-chemical properties dipoles, and volumes of the side chains have been used. In paper [33], pseudoamino acid compositions have been used. Regardless of homology based approaches, in paper [34], a Gaussian Naive Bayes based method has been proposed for the prediction of the DNA-binding proteins by using random forest for ranking the features. The wrapper-based feature selection using forward best first search strategy was used for selecting the features. These features include the information from primary sequence, predicted secondary structure, predicted relative solvent accessibility, and PSSM. The authors compared their result with decision tree, logistic regression,* k*-nearest neighbor, support vector machine with polynomial kernel, and support vector machine with radial basis function. It was observed by the authors of the paper that the proposed method outperformed five other classifiers.

The prediction of RNA binding residues is necessary for understanding the function and mechanism of biological activities involved in RNA-protein interactions. So for the prediction of RNA binding sites in paper [35] the authors have proposed a Naive Bayes classifier by using amino acid sequence and various features, such as relative accessible surface area, sequence entropy, hydrophobicity, secondary structure, and electrostatic potential. To improve the prediction performance the support vector machines based method has been proposed in papers [36, 37] to predict RNA binding sites by using the different sequential and structural features. In paper [36], sequence of amino acids and PSSM has been used. Then to improve the prediction performance in paper [37], the authors used smoothed PSSM with the correlation and dependency from the neighboring residues for each amino acid in a protein. A Naïve Bayes classifier with support vector machine has been proposed in paper [38] to predict RNA binding sites by using structural and topological information. The authors compared the results with different datasets, and it was observed by the authors that the highest AUC was achieved by a support vector machine, by using PSI-BLAST profile, accessible surface area, and retention coefficient. In paper [39] the authors have developed an enriched random forest based method with the features amino acid sequences such as PSSM, physicochemical properties of amino acids, polarity charge, and hydrophobicity to predict the RNA binding sites. In paper [40] the authors have used the majority voting system with protein sequence amino acid composition and physicochemical properties such as hydrophobicity, predicted secondary structure, predicted solvent accessibility, normalized Vander Waals volume, polarity, and polarizability for the prediction of RNA binding sites.

In paper [41] the authors have proposed integrated SVMs based method for the prediction of rRNA, RNA, and DNA-binding proteins by using protein sequence amino acid composition and physicochemical properties such as hydrophobicity, predicted secondary structure, predicted solvent accessibility, normalized Vander Waals volume, polarity, and polarizability. In papers [19, 42] the authors have developed a support vector machines based method for the prediction of DNA and RNA binding residues by using three amino acid sequence features. In paper [19], side chain pKa value, hydrophobicity index, and molecular mass have been used. In paper [42], the authors used PSSM with mean and standard deviation of side chain pKa value, hydrophobicity index, and molecular mass. It was observed by the authors that sensitivity and specificity were increased by 8% by using PSSM with mean of all three features. In paper [44], the authors have proposed the Bayesian classifier based method for the recognition of zinc binding sites of protein by using structural properties of a protein. In paper [43] the SVM based approaches have been proposed to predict metal binding by using the sequence and structural properties of proteins. In paper [45] a knowledge based method has been proposed with the combination of structural comparison and the evaluation of statistical potential for identifying DNA-binding proteins and binding sites. In paper [46] an approach based on binding assessment with distance-scaled, finite, ideal gas reference based statistical energy function and structural alignment of known protein has been proposed for the simultaneous prediction of RNA binding proteins and binding sites.  Table 1 presents the summary of various computational intelligence techniques in prediction of binding sites.

### 2.2. Computational Intelligence Techniques in Prediction of Subcellular Location

The subcellular location of a protein is closely correlated to the function of the protein. So it is necessary to design a robust and efficient computational intelligence techniques based method to predict subcellular locations in a protein. So for achieving this objective various computational intelligence techniques have been proposed in literatures. Some of the prominent computational intelligence techniques reported in literature for the application under consideration include artificial neural network (ANN), support vector machine (SVM), and* k*-nearest neighbors (*k*-NN). This section of the paper presents an analysis of various research papers in literature and examines the efficacy of each of these methods for the predictions of subcellular locations in a protein which are as follows.

The SVM based methods have been proposed in papers [47–51] to predict protein subcellular location by using different sequence derived properties. In paper [47] amino acid compositions have been used. In paper [48] the functional domain compositions of protein have been used. In paper [49], amino acid subsequences have been used. In paper [50], physiochemical properties of amino acid have been used. In paper [51], amino acid and dipeptide composition have been used. It is observed that physiochemical properties are much useful to predict the subcellular localization. But to increase the prediction performance in paper [52] a hybrid approach based method have been proposed that integrates PSI-BLAST and three SVM modules based on compositions of residues, dipeptides, and physicochemical properties to predict the subcellular localization of gram-negative bacterial proteins.

The* k*-nearest neighbor based methods has been proposed by the authors of papers [53–56] to improve the prediction performance of subcellular locations with different sequence derived properties. In paper [53], dipeptide composition of amino acids has been used. In paper [54], amino acid compositions, dipeptide compositions, and physicochemical properties have been used. In paper [55], functional domain composition has been used. In paper [56], low dimensional feature vector that is represented by fusing PSSM and pseudoamino acid composition has been used. Here it is observed that the PSSM with physiochemical properties may provide better results.

In paper [57] the authors have proposed a SVM based approach for the prediction of protein subcellular localization by using the integration of N-terminal targeting sequences amino acid composition and protein sequence motifs. In paper [58] the authors have proposed the C-support vector machine with pseudoamino acid composition. In this paper authors used multiscale energy to extract the features which provide the information about the sequence order to predict protein subcellular locations. In paper [59] the authors have proposed an evolutionary SVM by using combination of genetic algorithm with SVM to select most appropriate feature of physiochemical composition by using physiochemical property of amino acid to predict subcellular locations in a protein.

In papers [60–63] the authors have used support vector machine with different sequence derived properties, sequence motifs, and alignments to predict the subcellular locations of a protein. In paper [60], the combination of sequence alignment and amino acid composition has been used. In paper [61], amino acid composition and PSSM have been used. In paper [62], pseudoamino acid composition has been used. In paper [63], sequence motifs including motifs with gap have been used to predict the subcellular locations of a protein. In paper [64] the authors have developed a SVM and adaptive neurofuzzy based system by using amino acid composition, amino acid pair, 1-gapped-amino-acid pair, 2-gapped-amino-acid pair, and 3-gapped-amino-acid pair compositions. It was observed by the authors that the proposed method performed better in comparison to previous proposed methods. In paper [65] the authors have proposed a SVM based prediction system that integrates features from phylogenetic profiles and gene ontology (GO) terms derived from the protein sequence to improve the prediction performance of subcellular locations in a protein.

In paper [66] the authors have proposed a recurrent neural network based method by using the essential features that is extracted by principal component analysis (PCA) from amino acid composition. Similarly in paper [67] the authors have proposed an N-to-1 neural network with protein sequence for the prediction of subcellular location of proteins. In paper [68] the authors have proposed a SVM based approach along with a diversity algorithm by using amino acid composition, dipeptide, composition, reduced physiochemical properties, gene ontology, evolutionary information, and pseudoaverage chemical shift for the prediction of submitochondria location. In paper [69] the authors have proposed a hybrid method that used a support vector machine and an artificial neural network with known locations and structure of a protein for the prediction subcellular localization of proteins.  Table 2 presents a summary and performance evaluation of various computational intelligence techniques used for prediction of subcellular localization of a protein.

### 2.3. Computational Intelligence Techniques in the Prediction of Enzyme Function/Family

Enzyme catalyzes biochemical reactions and plays a very important role in the metabolic pathways. So it is necessary to design a robust and efficient computational intelligence techniques based method to predict enzyme function. So for achieving this objective various computational intelligence techniques have been proposed in literatures. Some of the prominent computational intelligence techniques reported in literature for the application under consideration include random forest, artificial neural network (ANN), support vector machine (SVM), and* k*-nearest neighbors (*k*-NN). This section of the paper presents an analysis of various research papers in literature and examines the efficacy of each of these methods for the predictions of enzyme function which are as follows.

In paper [145] the authors have developed an artificial neural network based method for the classification of enzymes from sequence by using sequence similarity and other sequence derived features such as cotranslational and posttranslational modification, secondary structure, and physical and chemical properties. The* k*-nearest neighbor based methods have been proposed by the authors of papers [70–74] by using different sequence derived properties. In paper [70], functional domain composition of a protein has been used. In paper [71], pseudoamino acid composition that includes both features such as sequence order related features and the function related features has been used. In paper [72], functional domain composition and PSSM have been used. In paper [73], amino acid composition has been used. In paper [74], pseudoamino acid composition with approximate entropy and the hydrophobicity pattern of an amino acid sequence have been used to predict enzyme function of a protein.

The SVM based methods have been proposed by the authors of papers [20, 75–82] by using different sequence derived properties. In paper [75], pseudoamino acid composition has been used. In paper [76], hydrophobicity of amino acid from pseudoamino acid composition has been used. In papers [77, 79], pseudoamino acid composition with the conjoint triad features (CTF) to represent the protein sequences not only the composition of amino acids, but also the neighbor relationships in the sequence have been used. In paper [78], feature vector from protein functional domain composition has been used. In paper [20], amino acid sequence has been used. In paper [80], features extracted from the global structure based on fragment libraries have been used. In paper [81], pseudoamino acid composition has been used. In paper [82], amino acid composition, physiochemical properties, and dipeptide composition have been used to predict enzyme functions of a protein. It is observed that the pseudoamino acid composition with the conjoint triad features provides better results than other features. In paper [83] the authors have proposed the Bayesian based approach with structure derived properties of a protein to predict the function of an enzyme.

The random forest based method has been proposed by the authors of papers [84, 85] to predict the functional class and subclass of enzymes by using sequence derived features. In paper [84], the authors have proposed a top-down three-layer approach where the top layer classified a query protein sequence as an enzyme or nonenzyme, the second layer predicted the main function class, and bottom layer further predicted the subfunction class. In paper [85], the authors have used a set of specificity determining residues to predict the class and subclass of an enzyme. In paper [86], the authors have proposed a SVM and random forest based methods by using sequence derived properties to predict the enzyme function and subfunctions. In paper [87], the authors have proposed an N-to-1 Neural Network for accurate prediction of enzyme by using amino acid sequences. It is observed that random forest based proposed methods with sequence derived properties provide the better results so random forest is much useful to predict the enzyme function and subfunctions.  Table 3 presents a summary and performance evaluation of various computational intelligence techniques used for prediction of enzyme function/family.

### 2.4. Computational Intelligence Techniques in Prediction of Signal Peptide

A signal peptide is a small peptide that is anticipated towards the secretory pathway. So it is necessary to design a robust and efficient computational intelligence techniques based method to predict the signal peptide. So for achieving this objective various computational intelligence techniques have been proposed in literatures. Some of the prominent computational intelligence techniques reported in literature for the application under consideration include artificial neural network (ANN) and* k*-nearest neighbors (*k*-NN). This section of the paper presents an analysis of various research papers in literature and examines the efficacy of each of these methods for the predictions of signal peptides which are as follows.

The ANN based method has been proposed by the authors of papers [88, 89] to predict the signal peptide by using amino acid sequences. Similarly in paper [90], the authors have proposed a bidirectional recurrent neural network based approach for the prediction of signal peptides in human protein sequences. In paper [92], the authors have proposed a neural network based method for detection of signal peptides in proteins by using the divided protein sequence into overlapping short sequence fragments. Then each fragment was analyzed with respect to the probability of it being a signal peptide. In paper [91], the authors have proposed an ensemble classifier that was formed by fusing many individual optimized evidences theoretic* k*-nearest neighbors for the prediction of signal peptide sequences and their cleavage sites by using pseudoamino acid composition. In papers [93, 94], authors have proposed a support vector machine based method for predicting signal peptides and their cleavage sites. In paper [93], pseudoamino acid composition has been used. In paper [94], position specific amino acid composition has been used. In paper [95] the authors have proposed a Bayesian reasoning network that was formed by fusing the results of different Bayesian classifiers which used sequence derived features through the weighted voting system based method to predict the N-terminal signal peptide and cleavage site.  Table 4 presents a summary and performance evaluation of various computational intelligence techniques used for prediction of signal peptides.

### 2.5. Computational Intelligence Techniques in Prediction of Catalytic Residue

The enzyme active site is the binding site for catalytic reactions of enzymes. So it is necessary to design a robust and efficient computational intelligence techniques based method to predict the catalytic residues. So for achieving this objective various computational intelligence techniques have been proposed in literatures. Some of the prominent computational intelligence techniques reported in literature for the application under consideration include artificial neural network (ANN) and support vector machine (SVM). This section of the paper presents an analysis of various research papers in literature and examines the efficacy of each of these methods for the predictions of catalytic residues which are as follows.

In paper [96] the authors have proposed a neural network based approach for the prediction of catalytic residues by using protein sequence and structural properties. Similarly in paper [97] the authors have proposed an integrated genetic algorithm and neural network based method for the prediction of catalytic residues by using residue properties. The support vector machine based method has been proposed by the authors of papers [98–101] by using different sequence derived properties. In paper [98], protein sequence and structural properties have been used. In paper [99], protein structure has been used. In paper [100], sequence and structural features have been used. In paper [101], structural features of a protein have been used for the prediction of catalytic residues.  Table 5 presents a summary and performance evaluation of various computational intelligence techniques used for prediction of catalytic residue.

### 2.6. Computational Intelligence Techniques in Prediction of Nuclear/G-Protein Coupled Receptor

Nuclear receptors (NR) are key transcription factors that regulate a wide variety of biological processes, such as homeostasis, reproduction, development, and metabolism. The nuclear receptors are involved in many physiological and pathological processes so prediction of different NR families and subfamilies is a most challenging problem in bioinformatics. The G-protein coupled receptors (GPCR) are involved in various physiological processes. So it is necessary to design a robust and efficient computational intelligence techniques based method to predict the different NR and GPCR subfamilies. So for achieving this objective various computational intelligence techniques have been proposed in literatures. Some of the prominent computational intelligence techniques reported in literature for the application under consideration include support vector machine (SVM) and* k*-nearest neighbors (*k*-NN). This section of the paper presents an analysis of various research papers in literature and examines the efficacy of each of these methods for the predictions of NR and GPCR subfamilies which are as follows.

In papers [102–106] the authors have proposed the SVM based methods by using different sequence and structural features. In paper [102], amino acid composition and dipeptide of amino acids have been used. In paper [103], 4-tuple residue composition instead of dipeptide composition to encode the sequences has been used. In paper [104], pseudoamino acid composition and, similarly in paper [105], pseudoamino acid composition whose components were derived from a physical-chemical matrix via a series of autocovariance and cross-covariance transformations have been used. In paper [106], amino acid composition, dipeptide composition, and physicochemical properties have been used to predict the nuclear receptor and their subfamilies. In paper [107], the authors have proposed a fuzzy* k*-nearest neighbor classifier based on the pseudoamino acid composition with physicochemical and statistical features derived from the protein sequences, such as amino acid composition, dipeptide composition, complexity factor, and low-frequency Fourier spectrum components to predict the nuclear receptors and their subfamilies. Here it is observed that amino acid composition, dipeptide composition with pseudoamino acid compositions may provide the better results.

In papers [21, 108], the authors have proposed the SVM based method to predict the G-protein coupled receptors and their subfamilies. In paper [108], dipeptide composition of amino acids and, similarly in paper [21], amino acid and dipeptide composition have been used. A nearest neighbor method has been proposed in paper [109] to discriminate GPCRs from non-GPCRs. In this paper, GPCRs have been classified at four levels on the basis of amino acid composition and dipeptide composition of proteins. In paper [110], the authors have proposed an adaboost classifier to predict G-protein coupled receptors by using pseudoamino acid composition with approximate entropy and hydrophobicity patterns. In paper [111], the authors have proposed a principal component analysis based method for the prediction of GPCRs, families, subfamilies, subsubfamilies, and subtype. In this paper the authors used sequence derived features such as amino acid composition, dipeptide composition, autocorrelation descriptors, normalized Vander Waals volume, polarity, polarizability, charge, secondary structure, solvent accessibility, relative hydrophobicity, and pseudoamino acid composition. Here it is observed that amino acid composition, dipeptide composition, and pseudoamino acid compositions with physiochemical properties based methods may provide better results to predict the GPCR and their subfamilies.  Table 6 presents a summary and performance evaluation of various computational intelligence techniques used for prediction of nuclear/GPC receptor.

### 2.7. Computational Intelligence Techniques in Prediction of Membrane Protein

Membrane proteins are involved in various cellular processes to perform various important functions. So it is necessary to design a robust and efficient computational intelligence techniques based method to predict the membrane proteins. So for achieving this objective various computational intelligence techniques have been proposed in literatures. Some of the prominent computational intelligence techniques reported in literature for the application under consideration include support vector machine (SVM), ensemble classifiers, and* k*-nearest neighbors (*k*-NN). This section of the paper presents an analysis of various research papers in literature and examines the efficacy of each of these methods for the predictions membrane proteins which are as follows.

In papers [112, 113], the authors have proposed the* k*-nearest neighbor based method to predict the membrane proteins. In paper [112], the dimensionality reduction has been used to decrease the complexity. In this paper the original high-dimensional feature vectors transformed into the low dimensional feature vectors. Then this encoded sequence fused with PSSM and pseudoamino acid composition has been used. In paper [113], sequence homology and protein-protein interaction network with pseudoamino acid composition were used to predict the membrane proteins. In paper [114], the authors have proposed a fuzzy* k*-nearest neighbor algorithm by using pseudoamino acid composition to predict the membrane proteins. In paper [22], the authors have proposed an ensemble classifier formed by fusing many individual optimized evidence theoretic* k*-nearest neighbors and their types by using pseudoamino acid composition to predict the membrane proteins. A support vector machine based method has been proposed in paper [115] for the classification of transmembrane proteins by using protein sequence. In paper [116], the authors have proposed the stepwise discriminant analysis for extracting high order sequence information for amino acids and peptides that were distinct for different types of the membrane proteins. Then their occurrence frequencies in membrane proteins have been used to predict the types of membrane proteins. Similarly in paper [117], the authors have proposed an ensemble classifier based on the pseudoamino acid composition and the approximate entropy to predict the types of membrane protein. Here it is observed that ensemble classifiers with pseudoamino acid compositions and PSSM may provide better results.  Table 7 presents a summary and performance evaluation of various computational intelligence techniques used for prediction of membrane protein.

## 3. Computational Intelligence Techniques in Protein Functions Prediction by Protein Interaction Network

Performing a specific function a protein must interact with another protein. The interaction of the protein is represented in the form of network called protein-protein interaction network. So by using the knowledge of this interaction network various computational techniques based approaches have been proposed for protein function prediction by using one or more interaction networks. These are categorized in four ways, the one that assigns a level to an annotated protein by transferring labels in its neighbor is known as neighbor based techniques, the second finds density connected region in the interaction network called cluster and assigns a label to an annotated protein based on most dominant label in the corresponding cluster is known as clustering based approaches, the third that utilizes the entire connectivity structure on the network is called optimization based technique, and the fourth that uses association analysis algorithm to detect frequently occurring sets in interaction network for protein function prediction is known as association analysis based techniques. In paper [146], the authors have proposed a graph based approach for global alignment of protein-protein interaction network based on alignment scoring matrix derived from both biological and topological information from the network. In this paper an alignment score matrix has been constructed by similarity score matrix and interaction score matrix. The similarity score matrix was the sum of topological score matrix and the biological score matrix that indicate the topological and biological similarity between two nodes in the protein-protein interaction networks, respectively. In paper [147], the authors have used a SVM and genetic algorithms for the detection of gene-gene interaction. In paper [148], the authors have developed a PCA based clustering algorithm to reduce the dimensionality and produce a better informative cluster for multiple functional association of protein. In paper [118], authors have proposed a Markov random field based approach for protein function prediction from protein-protein interaction network using functional probability of each protein by using a Bayesian approach. In paper [119] the authors have proposed a network flow based algorithm for exploiting the protein-protein interaction network. A hyper clique pattern is a type of an association pattern which contains protein and is highly associated with each other. Proteins within the same hyper clique pattern more likely perform the same function and participate in the same biological process. Therefore in paper [149], the authors have proposed a hypercube pattern discovery approach for extracting functional module (hyper clique pattern) for protein function prediction. In paper [120], the authors have developed two-step algorithm to predict the protein function. In this paper the authors first assigned a weight to each of level 1 and level 2 neighbors by calculating its functional similarity with the protein using the protein-protein interaction network and then scoring each function based on the weighted frequency in the neighbors. Similarly in paper [121] authors have proposed an association analysis based method by using* h*-confidence to find exact group of object having high similarity with each other. In paper [122] the authors have proposed a Naïve Bayes approach by using information from protein-protein interaction network to predict protein function. Genes with similar functions have similar annotation patterns in their neighborhood, regardless of the distance between them in the interaction network. Therefore in paper [123], the authors have proposed a two-phase approach to predict molecular functions of uncharacterized genes by comparing their functional neighborhoods to genes of known function. In this paper authors have extracted functional neighborhood features of a gene using random walks with restarts (RWR). Then authors used a* k*-NN classifier to predict the function of uncharacterized genes based on the computed neighborhood features. In paper [124], the authors have proposed a method for protein function prediction by integrating the gene expression data and protein-protein interaction data. In this paper the authors considered the dynamic value of protein-protein interaction network and constructed the time sequenced subnetwork according to the time when the network was activated. Then authors have developed an algorithm to identify the protein complexes from time sequenced subnetwork and then applied a second algorithm to predict the protein function from these protein complexes from the time sequenced subnetwork. In paper [125], the authors have used the graph based centrality metrics to select proper candidate for labeling. Firstly the authors clustered a PPI network by using spectral clustering algorithms and selected a proper candidate for labeling within each cluster. Then they applied a collective classification for protein function prediction. In paper [126] the authors have proposed a multilevel classification technique by using function-function correlation to predict protein function from protein-protein interaction network. The previous proposed methods have used the shortest path distance as a measure proximity which has limited ability to capture fined grained neighborhood distinction because most of the protein is closed to each other and there are many ties in proximity. So by considering this problem in paper [127], the authors have proposed a diffusion state distance to capture a fined grained distinction in proximity for transfer of function prediction in protein-protein interaction network. It is observed that the neighbor based, association analysis based, graph based, and clustering based methods are useful in protein function prediction by using protein-protein interaction network but integration of PPI data with gene expression data may provide better results.  Table 8 presents a summary and performance evaluation of various computational intelligence techniques for protein function prediction by using protein interaction network.

## 4. Computational Intelligence Techniques in Protein Functions Prediction by Gene Expression Data

Gene expression is the process by which information from a gene is transformed into functional product such as protein or RNA by transcription and translation process. DNA microarrays are used to analyze the gene expression level. Gene expression data are analyzed in the form of a matrix where each row represents a gene and each column represents a sample. Gene expression data first filtered and normalized before using cluster analysis. So it is necessary to design a robust and efficient computational intelligence techniques based method to predict the protein function by using gene expression data. So for achieving this objective various computational intelligence techniques have been proposed in literatures. Some of the prominent computational intelligence techniques reported in literature for the application under consideration include support vector machine (SVM), genetic programming,* k*-means, self-organizing map (SOM), and Hypergraph. This section of the paper presents an analysis of various research papers in literature and examines the efficacy of each of these methods to predict the protein function by using gene expression data which are as follows.

In papers [150–154] The self-organizing map (SOM) has been used for the analysis of gene expression data. In paper [128] the authors have proposed a multilayer perceptron for gene function prediction from gene expression data. In paper [129] an integrated probabilistic model has been proposed that combined protein physical interactions, genetic interactions, highly correlated gene expression network, protein complex data, and domain structures of individual proteins together for the prediction of protein functions. In paper [130] a multiclass support vector machine has been developed for the cancer diagnosis from gene expression data.

In papers [131–134] the genetic programming based methods have been proposed for the classification of gene expression data. In paper [131], the authors have used the similarity between classification rules by matching in representation level and then a set of comprehensive and precise rules was obtained by genetic programming after evaluating the diversity. Then a fusion based method has been used with a subset of diverse classification rules for the final decision. In paper [132], the authors have proposed a majority voting technique for prediction of the labels of test samples. In this paper the authors have evolved multiple rules instead of a single set of rules with genetic programming and then applied those rules to test samples to determine their labels by using the majority voting technique. In papers [133, 134], genetic programming based approaches have been proposed for the classification of gene expression datasets.

In paper [135] the authors have proposed a fuzzy nearest clusters method for gene function prediction by using gene expression data. In this paper the hierarchical clustering has been used to detect homogeneous coexpressed gene subgroups or clusters in each possible heterogeneous functional class. Then classification algorithm was used to predict the functional roles of the unclassified genes based on their corresponding similarities to the detected functional clusters. In paper [136] the authors have used a* k*-means method for clustering gene expression data by incorporating protein-protein interaction data to improve the similarity measures. In paper [137] an unnormalized, random walk and symmetric normalized hyper graph, which is a Laplacian based semisupervised learning method, have been proposed for the gene function prediction by using gene expression data. In paper [138] the authors have proposed a discriminative local subspace that combines supervised machine learning and coexpression technique for the gene function prediction.  Table 9 presents a summary and performance evaluation of various computational intelligence techniques for protein function prediction by using gene expression data.

## 5. Computational Intelligence Techniques in Pathway Analysis from Gene Expression Data

The pathway is a series of interconnected enzymatic steps linked with the production of intermediates that are used in the next enzymatic step so we can say that it is a series of consecutive enzymatic reactions that produce specific products. Pathway consists of genes that chemically act together for specific cellular or physiologic function so pathway analysis is useful for gene function prediction. There are two types of pathways, metabolic pathways and signaling pathways. The metabolic pathways are biological network that involves enzymatic catalysis, while signaling pathways are the series of specific action in a cell in which signal is passed in one molecule to the next in series. In pathway analysis each pathway will be ranked based on the score obtained either by the Enrichment analysis or by machine learning approaches. The highest score will be given to the pathway which has most relevant gene to related phenotype. So it is necessary to design a robust and efficient computational intelligence techniques based method for pathway analysis from gene expression data. So for achieving this objective various computational intelligence techniques have been proposed in literatures. Some of the prominent computational intelligence techniques reported in literature for the application under consideration include gene set enrichment analysis, linear discriminant analysis, random forest, and decision tree based ensemble classifiers. This section of the paper presents an analysis of various research papers in literature and examines the efficacy of each of these methods for pathway analysis from gene expression data which are as follows.

In paper [155] the authors have used the combination of a scoring function for the prediction of most interesting pathways from gene expression data. In paper [139] a gene set enrichment analysis (GSEA) based method has been proposed for pathway analysis by using gene expression data with significance analysis of microarray. In paper [140] the authors have proposed a LDA based method for the classification of gene expression data by integrating biological knowledge of gene functions. In paper [141] the random forest has been used for clustering the pathways. In this paper the authors have constructed the decision trees using a gene in a pathway and combined the decision tree of all the pathways into a forest to predict the phenotype. In paper [142] the authors have proposed a Naïve Bayes and Decision Tree based ensemble classifier for the prediction of metabolic pathways from gene expression data. In paper [143], a two-stage machine learning algorithm has been proposed for pathway analysis from gene expression data. Similarly in paper [144] the authors have proposed a hierarchical Bayesian model for the prediction of pathways by using gene expression data.  Table 10 presents a summary and performance evaluation of various computational intelligence techniques in pathway analysis from gene expression data.

## 6. Observations and Discussions

### 6.1. Computational Intelligence Techniques in Protein Function Prediction by Using Sequence and Structure

Some of the observations related to the computational intelligence techniques for predictions of DNA and RNA binding sites presented in Section 2.1 are as follows.

- The ANN, SVM, random forest, and Naïve Bayes based methods are useful for the prediction of DNA, RNA, and metal binding sites.
- The overall accuracy obtained by ANN ranges in between 64% and 73.6%, ranges in between SVM 77% and 96.6%, and ranges in between random forest 78% and 91.41% and Naïve Bayes ranges in between 79% and 85% for the various diverse datasets as reported in Table 1.
- The SVM based method obtained maximum 96.6% by using various sequence derived properties and random forest obtained maximum 91.41% accuracy by using various sequence and structural properties for the prediction of DNA binding sites.
- The SVM based method obtained maximum 87.99% by using various sequence derived properties with PSSM and random forest obtained maximum 88.63% accuracy by using various sequence derived properties with PSSM for the prediction of RNA binding sites.

Therefore, from the above observations it is recommended that the combination amino acid composition, dipeptide composition, pseudoamino acid composition, correlation factors, and PSSM with support vector machine may be useful for the prediction of DNA and RNA binding sites.

Some of the observations related to the computational intelligence techniques in prediction of subcellular locations of protein presented in Section 2.2 are as follows.

- The SVM,* k*-NN, and ANN based methods are useful for the prediction of subcellular localization of protein.
- The overall accuracy obtained by SVM ranges in between 66.7% and 94%, ANN ranges in between 68% and 89%, and* k*-NN ranges in between 80% and 93.57% for the various diverse datasets as reported in Table 2.
- The SVM based method obtained maximum 94% accuracy by using amino acid composition, amino acid pair, and 1, 2, 3 gapped amino acid pair compositions.
- The* k*-NN based method obtained maximum 93.57% accuracy by using PSSM and pseudoamino acid composition.
- The combination of SVM and ANN based method obtained only 68% accuracy by using structural properties of a protein for the prediction of subcellular localization.
- So from the analysis it is observed that the only sequential properties of a protein affect the prediction performance of subcellular localization in comparison to structural properties.

Therefore from the above observations it is recommended that the* k*-NN based approaches by using combination amino acid composition, pseudoamino acid composition, PSSM, and physiochemical properties of sequence may be useful for the prediction of subcellular locations of protein.

Some of the observations related to the computational intelligence techniques in prediction of enzyme functions/families of protein presented in Section 2.3 are as follows.

- The SVM, random forest, and* k*-NN based methods are useful for the prediction of enzyme functions and families.
- The overall accuracy obtained by SVM ranges in between 69.1 and 99.53%, random forest ranges in between 71.29 and 99.31%, and* k*-NN ranges in between 56.9 and 99.0% for the various diverse datasets as reported in Table 3.
- All the SVM, random forest, and* k*-NN based method obtained maximum accuracy by using the sequence derived properties.
- The variation of ANN is proposed as N-to-1 neural network and by using protein sequence and obtained 96% accuracy.
- So from the analysis it is observed that the sequence derived properties are useful to predict the enzyme functions and families.

Therefore from the above observations it is recommended that the random forest based ensemble classifier by using combination amino acid composition, dipeptide composition, and pseudoamino acid composition with physiochemical properties of sequence may be useful for the prediction of enzyme functions/families of protein.

Some of the observations related to the computational intelligence techniques in prediction of signal peptides of protein presented in Section 2.4 are as follows.

- The ANN and SVM based methods are useful for the prediction of signal peptides.
- The overall accuracy obtained by ANN ranges in between 93% to 97%, and the same obtained by SVM ranges in between 90.97 to 97% for the various discrete datasets as reported in Table 4.
- Both the ANN and SVM based methods obtained maximum 97% accuracy by using amino acid sequence and pseudoamino acid composition, respectively.
- Bayesian reasoning network obtained 97.73% accuracy by using sequence derived properties; thus it is also useful to predict the signal peptides.

Therefore from the above observations it is recommended that the SVM and based methods by using sequence derived properties may be useful for the prediction of signal peptides in protein.

Some of the observations related to the computational intelligence techniques in prediction of catalytic residues of protein presented in Section 2.5 are as follows.

- The ANN and SVM based methods are useful for the prediction of catalytic residues.
- The overall accuracy obtained by ANN ranges in between 69 and 91.2% and for SVM ranges in between 86 and 95.76% (see Table 5).
- The ANN based method obtained maximum 91.2% accuracy by using the residue properties of protein sequence.
- The SVM based method obtained maximum 95.76% accuracy by using the sequence and structural properties of protein.

Therefore from the above observations it is recommended that the SVM and based methods by using sequence and structural derived properties may be useful for the prediction of catalytic residues of protein.

Some of the observations related to the computational intelligence techniques in prediction of nuclear and G-protein coupled receptors of protein presented in Section 2.6 are as follows.

- The SVM and fuzzy* k*-NN based methods are useful for the prediction of nuclear and G-protein coupled receptors families and their subfamilies.
- The overall accuracy obtained by SVM ranges in between 82.8 and 99.6% and by fuzzy* k*-NN is 93% (see Table 6).
- The fuzzy* k*-NN based method obtained maximum 93% overall accuracy by using the pseudoamino acid composition with physicochemical and statistical features.
- The SVM based method obtained maximum 99.6% accuracy by using the pseudoamino acid composition of protein.

Therefore from the above observations it is recommended that the SVM based methods by using amino acid composition and dipeptide composition with pseudoamino acid compositions may be useful for the prediction of nuclear and G-protein coupled receptors families and their subfamilies.

Some of the observations related to the computational intelligence techniques in prediction of membrane protein presented in Section 2.7 are as follows.

- The SVM, ensemble classifier, and* k*-NN based methods are useful for the prediction of membrane proteins.
- The overall accuracy obtained by SVM 90.1%, ensemble classifier 91.2%, and* k*-NN based classifier ranges in between 87.65 and 95.7% (see Table 7).
- The fuzzy* k*-NN based method obtained maximum accuracy of 95.7% by using the pseudoamino acid composition.
- The ensemble based classifier obtained maximum accuracy of 91.2% by using the pseudoamino acid composition with approximate entropy.

Therefore from the above observations it is recommended that the fuzzy* k*-NN based methods pseudoamino acid compositions and PSSM with approximate entropy may be useful for the prediction of membrane portions.

### 6.2. Computational Intelligence Techniques in Protein Function Prediction by Protein Interaction Network

Some of the observations related to the Computational Intelligence Techniques in Protein Function Prediction by Protein Interaction Network presented in Section 3 are as follows.

- The neighbor based, association analysis based, graph based, and clustering based methods are useful in protein function prediction by using protein-protein interaction network.
- A graph based approach is useful for global alignment of protein-protein interaction network by using alignment scoring matrix.
- SVM and genetic algorithms are useful for the detection of gene-gene interaction.
- The network flow based method obtained maximum 90% accuracy by using protein interactions maps.
- The neighbor based method obtained maximum 98% accuracy by using both label 1 and label 2 neighbors.
- The association based method obtained maximum 93% accuracy by using* h*-confidence and adjacency matrix.
- The integration of gene expression with protein-protein interaction data is also useful for protein function prediction.

Therefore from the above observations it is recommended that neighbor based methods by using the protein-protein interaction data such as diffusion state matrix,* h*-confidence values, alignment score matrix may be useful for protein function prediction.

### 6.3. Computational Intelligence Techniques in Protein Function Prediction by Gene Expression Data

Some of the observations related to the Computational Intelligence Techniques in Protein Function Prediction by gene expression data presented in Section 4 are as follows.

- The genetic programming, SVM, hypergraph, and clustering based approaches are useful in function prediction by using gene expression data.
- The overall accuracy obtained by genetic programming ranges in between 81.82 and 100%, clustering based methods ranges in between 16 and 65.27%, SVM 89.44% and by hypergraph based method 97.95% (see Table 9).
- It is also observed that the genetic programming with gene expression data provides better results.

Therefore from the above observations it is recommended that genetic programming based methods by using the gene expression data with constant values may be useful for protein function prediction.

### 6.4. Computational Intelligence Techniques in Pathway Analysis from Gene Expression Data

Some of the observations related to the Computational Intelligence Techniques in Protein Function Prediction by gene expression data presented in Section 5 are as follows.

- The gene set enrichment analysis (GSEA), linear discriminant analysis, Naïve Bayes, and decision tree based ensemble classifiers are useful in pathways analysis of gene expression data.
- The overall accuracy is obtained by GSEA 78%, by Naive Bayes and decision tree based ensemble classifier 91.2%, and by Bayesian approach 75.7% (see Table 10).
- It is observed that the maximum accuracy is observed by Naive Bayes and decision tree based ensemble classifier.
- The linear discriminant analysis based method provides 11–17% error rate by using covariance matrix with group relationship among variables while SVM, Bayesian approach, and C5.0 with random forest based method provide 7–15% error rate.

Therefore from the above observations it is recommended that SVM, Naive Bayes, and C5.0 with random forest based methods by using the gene expression data with covariance matrix with group relationship among variables may be useful for protein function prediction.

## 7. Case Study for Protein Function Prediction by Using Sequence Derived Properties

The paper shows that there are various computational intelligence based methods that are used to predict the protein function using sequence, structure, protein-protein interaction network, and gene expression data. The papers reported in literature used different-different data to predict the protein function. Therefore for the comparative analysis of computational intelligence based approaches a protein sequence of different functions is collected from UniProt database. For the classification of various protein functions 384 numbers of DNA binding sites, 136 numbers of RNA binding sites, 463 numbers of membrane protein, 522 numbers of enzymes, 384 numbers of nuclear receptors, and 384 numbers of G-protein coupled receptors of sequences have been collected from the UniProt database. For the prediction of protein function three feature vectors with 20 numbers of amino acid composition, 400 numbers of dipeptide composition, and 50 numbers of pseudoamino acid composition, thus a total of 470 numbers of features, are calculated. The support vector machine,* k*-nearest neighbor classifier, random forest, and Naïve Bayes are used for the prediction of protein function by using sequence derived properties of sequences. The 10-fold cross validation is used for the performance analysis of the classifiers. The complete results analysis is shown in Table 11.

The results of Table 11 show that the performance of support vector machine is better in comparison with other classifiers to predict the protein function from sequence derived features. The support vector machine based method provides overall accuracy of 84.1% and MCC values of 0.82 with amino acid composition (see Table 11). The overall accuracy increases from 84.1% to 91% and from 91% to 91.5% by using dipeptide and pseudoamino acid composition, respectively (see Table 11). Therefore it is observed from Table 11 that the fusion of sequence derived properties improves the prediction performance of the classifier to predict the protein function perdition.

## 8. Conclusion

This paper presented a state-of-the-art comprehensive review of various computational intelligence techniques for protein function predictions using sequence, structure, protein-protein interaction network, and gene expression data used in wide areas of applications such as prediction of DNA and RNA binding sites, subcellular localization, enzyme function, signal peptide, catalytic residue, nuclear/G-protein coupled receptor, membrane protein, and pathway analysis from gene expression datasets. The summaries of the results obtained by many researchers to solve these problems by using computational intelligence techniques with appropriate datasets to improve the prediction performance have been presented. The summaries presented in this paper indicate that ensemble classifiers and integration of multiple heterogeneous data are also useful for protein function prediction. The most successful approach is the integration of multiple computational intelligence techniques with integrating the multiple heterogeneous data to predict protein function. However, there are more possibilities for improvement in this area, since there are large numbers of proteins available with unknown function.

## Figures and Tables

**Table 1:** Summary of computational intelligence (CI) techniques in prediction of binding sites.

Reference	CI techniques	Binding sites/residues	Performance	Datasets
[23]	ANN	DNA	Accuracy: 64%, sensitivity: 69%	Amino acid sequence composition, solvent accessibility, and secondary structure

[24]	ANN	DNA	Accuracy: 73.6%	Position specific scoring matrices (PSSM)

[25]	SVM	DNA	Accuracy: 90%	Surface and overall composition, overall charge and positive potential patches on the protein surface

[26]	SVM	DNA	Accuracy: 82.30%	Amino acid sequence, PSSM, and low-resolution structural information

[18]	SVM	DNA	Accuracy: 77.2%, sensitivity: 76.4%, and specificity: 76.6%	Position specific scoring matrices (PSSM)

[27]	SVM	DNA	Accuracy: 96.6%, sensitivity: 90.7%	Amino acid sequence, pseudoamino acid composition, autocross-covariance transforms, and dipeptide composition

[28]	SVM	DNA	Accuracy: 80%, sensitivity: 85.1%, and specificity: 85.3%	Normalized PSSM score, normalized solvent accessible surface area, and protein backbone structure

[29]	SVM	DNA	MCC: 0.67, accuracy: 89.6%, sensitivity: 88.4%, and specificity: 90.8%	PSSM, amino acid composition, hydrophobicity, polarity, polarizability, secondary structure, solvent accessibility, normalized Vander Waals volume, binding propensity, and nonbinding propensity

[30]	Ensemble of ANN and SVM	DNA	Accuracy: 89.00%	PSSM and structural features such as secondary structure, solvent accessibility, and globularity

[31]	Random forest	DNA	Accuracy: 78.20%, sensitivity: 78.06%, and specificity: 78.22%	PSSM with mean and standard of deviation side chain pKa value, hydrophobicity index, and molecular mass

[32]	Random forest	DNA	Accuracy: 91.41%, MCC: 0.70, and AUC: 0.913	PSSM, secondary structure information, and orthogonal binary vector information and two physical-chemical properties dipoles and volumes of the side chains

[33]	Random forest	DNA	Accuracy: 83.96%	Pseudoamino acid composition

[34]	Gaussian Naive Bayes	DNA	Accuracy: 79.10% and MCC: 0.583	PSSM, predicted secondary structure, predicted relative solvent accessibility

[35]	Naive Bayes classifier	RNA	Accuracy: 85.00%	Amino acid sequence, relative accessible surface area, sequence entropy, hydrophobicity, secondary structure, and electrostatic potential

[36]	SVM	RNA	MCC: 0.31	Amino acid sequence and PSSM

[37]	SVM	RNA	Accuracy: 87.99%, sensitivity: 79.95%, and specificity: 90.36%	Smoothed PSSM with the correlation and dependency from the neighboring residues

[38]	SVM	RNA	AUC: 0.83	PSSM, accessible surface area, between centrality and retention coefficient

[39]	Random forest	RNA	MCC: 0.5637, accuracy: 88.63%, sensitivity: 53.70%, and specificity: 96.97%	PSSM, physicochemical properties of amino acids, polarity-charge, and hydrophobicity

[40]	SVM	RNA	Accuracy: 79.72% and MCC: 0.59	Protein sequence, amino acid composition, hydrophobicity, secondary structure, predicted solvent accessibility, normalized Vander Waals volume, polarity, and polarizability

[41]	SVM	rRNA, RNA, and DNA	rRNA accuracy: 84%, RNA accuracy: 78%, DNA accuracy: 72%	Protein sequence, amino acid composition, hydrophobicity, secondary structure, predicted solvent accessibility, normalized Vander Waals volume, polarity, and polarizability

[19]	SVM	DNA and RNA	DNA sensitivity: 69.40%, specificity: 70.47%, and RNA sensitivity: 66.28%, and specificity: 69.84%	Side chain pKa value, hydrophobicity index and molecular mass

[42]	SVM	DNA and RNA	Accuracy: 79.00%, sensitivity: 77.30%, specificity: 79.30% for DNA, and accuracy: 77.70%, sensitivity: 71.60%, and specificity: 78.70% for RNA-binding residues	PSSM with mean and standard of deviation side chain pKa value, hydrophobicity index, and molecular mass

[43]	SVM	Metal binding	Accuracy: 78.10%	Physiochemical properties of the amino acid sequences

[44]	Bayesian classifier	Zinc	Specificity: 99.8%, sensitivity: 75.5%	Structural properties of a protein

[45]	Structural comparison	DNA	Accuracy: 98% and precision: 84%	Combination of structural comparison and the evaluation of statistical potential

[46]	Structural comparison	RNA	Accuracy: 98%, precision: 91% for predicting RBPs, and accuracy: 93% and precision: 78% for predicting RNA binding residues	Distance-scaled, finite, ideal gas reference based statistical energy function, and structural alignment

**Table 2:** Summary of computational intelligence (CI) techniques in prediction of subcellular localization.

Reference	CI techniques	Performance	Datasets
[47]	SVM	Accuracy: 86.3%	Amino acid compositions
[48]	SVM	Average accuracy: 66.7%	Functional domain composition of protein
[49]	SVM	Overall recall: 89.8%	Amino acid subsequence
[50]	SVM	Overall accuracy: 93.1%	Physiochemical property of amino acid
[51]	SVM	Accuracy: 84.9%	Amino acid composition, dipeptide composition, and similarity information
[52]	SVM	Accuracy: 91.2%	Compositions of residues, dipeptides, and physicochemical properties
[53]	*k*-NN	Overall accuracy: 80%	Dipeptide composition of amino acids
[54]	*k*-NN	Overall accuracy: 92.5%	Amino acid compositions, dipeptide compositions, and physicochemical properties
[55]	*k*-NN	Overall accuracy: 85.4%	Functional domain composition
[56]	*k*-NN	Overall accuracy: 93.57%	PSSM and pseudoamino acid composition
[57]	SVM	Overall accuracy: 74.00%	N-terminal targeting sequences amino acid composition and protein sequence motifs
[58]	CSVM	Overall accuracy: 80.03%	Pseudoamino acid composition
[59]	SVM with GA	Overall accuracy: 72.82%	Physiochemical property of amino acid
[60]	SVM	Overall accuracy: 90.96% and MCC: 0.8655	Combination of sequence alignment and feature based on amino acid composition
[61]	SVM	Accuracy: 73.71%	Amino acid composition and PSSM
[62]	SVM	Accuracy: 88.3%	Pseudoamino acid composition
[63]	SVM	Recall: 91.30%	Sequence motifs
[64]	SVM	Accuracy: up to 94.00%	Amino acid composition, amino acid pair, 1, 2.3 gapped amino acid pair compositions
[65]	SVM	Accuracy: up to 93%	Integrates features from phylogenetic profiles and gene ontology
[66]	Recurrent NN	Overall accuracy: 72.55%	Pseudo amino acid composition
[67]	N-to-1 NN	Accuracy: up to 89%	Protein sequence
[68]	SVM	Overall accuracy: 93.57%	Amino acid and dipeptide, composition, reduced physiochemical properties, gene ontology, PSSM, and pseudoaverage chemical shift
[69]	SVM and ANN	Accuracy: 68%	Structural properties of a protein

**Table 3:** Summary of computational intelligence (CI) techniques in prediction of enzyme function/family.

Ref.	CI techniques	Performance	Datasets
[70]	*k*-NN	Accuracy: 85%	Functional domain composition
[71]	*k*-NN	Accuracy: 76.6%	Amphiphilic pseudoamino acid composition
[72]	OET-*k*NN	Overall accuracy: 91.3%, 93.7%, and 98.3% for the 1st, 2nd, and 3rd level	Functional domain composition and PSSM
[73]	*k*-NN	Accuracy: 99%	Amino acid composition
[74]	Fuzzy *k*-NN	Accuracy: 56.9%	Pseudoamino acid composition, approximate entropy, and hydrophobicity
[75]	SVM	Accuracy: 80.87%	Amphiphilic pseudo amino acid composition
[76]	SVM with DWT	Accuracy: 91.9.	Pseudoamino acid composition
[77]	SVM	MCC: 0.92 and accuracy: 93%	Pseudoamino acid composition with CTF
[78]	SVM	Accuracy: 91.32%	Functional domain composition
[79]	SVM	Accuracy: 81% to 98% and MCC: 0.82 to 0.98	Pseudoamino acid composition with CTF
[20]	SVM	Accuracy: 95.25%	Structural features based on fragment libraries
[80]	SVM	Accuracy: 69.1–99.6%	Amino acid sequence
[81]	SVM	Sensitivity: 85.6% and specificity: 86.1%	Pseudoamino acid composition
[82]	SVM	Accuracy: 77.4%	Sequence similarity, amino acid composition, physiochemical properties, and dipeptide composition
[83]	Bayesian classifier	Accuracy: 45%	Structural properties
[84]	Random forest	Overall accuracy: 94.87%, 87.7%, and 84.25% for the 1st, 2nd, and 3rd level	Sequence derived features
[85]	Random forest	Precision: 0.98 and recall: 0.89	Set of specificity determining residues
[86]	SVM and random forest	Accuracy: 71.29–99.53% by SVM and 94–99.31% by random forest	Sequence derived properties
[87]	N-to-1 neural network	Overall accuracy: 96%, specificity: 80%, and FP rates: 7%	Amino acid sequences

**Table 4:** Summary of computational intelligence (CI) techniques in prediction of signal peptides.

Reference	CI techniques	Performance	Datasets
[88]	ANN	Accuracy: 97%	Amino acid sequences
[89]	ANN	Accuracy: 97%	Amino acid sequences
[90]	Bidirectional recurrent NN	Accuracy: 97%	Amino acid sequences
[91]	OET-*k*NN	Accuracy: 73.4%	Pseudoamino acid composition
[92]	ANN	Accuracy: 93%	Amino acid sequences
[93]	SVM	Accuracy: 97%	Pseudoamino acid composition
[94]	SVM	Sensitivity: 90.97% and selectivity: 97.42%	Position specific amino acid composition
[95]	Bayesian reasoning network	Accuracy: 97.73% for secretory and nonsecretory and 90.90% for signal peptide cleavage site	Sequence derived features

**Table 5:** Summary of computational intelligence techniques in prediction of catalytic residue.

Reference	CI techniques	Performance	Datasets
[96]	ANN	Accuracy: 69%	Features of amino acid sequence and structure
[97]	GA with ANN	Accuracy: 91.2%	Residue properties
[98]	SVM	Accuracy: 86%	Sequence and structural properties
[99]	SVM	Recall: 61%	Protein structure
[100]	SVM	Accuracy: 88.6%–95.76%	Sequence and structural properties
[101]	SVM	MCC: 0.74, sensitivity: 0.76, and specificity: 0.51	Structural features of a protein

**Table 6:** Summary of computational intelligence techniques in prediction of nuclear/GPC receptor.

Reference	CI techniques	Prediction	Performance	Datasets
[102]	SVM	NR	Overall accuracy: 82.6%–97.5%	Amino acid composition and dipeptide composition
[103]	SVM	NR	Overall accuracy: 96%	4-tuple residue composition
[104]	SVM	NR	Overall accuracy: 99.6%	Pseudoamino acid composition
[105]	SVM	NR	Accuracy: 98%	Pseudoamino acid composition
[106]	SVM	NR	Accuracy: 97%	Amino acid composition, dipeptide composition, and physicochemical property
[107]	Fuzzy *k*-NN	NR	Overall accuracy: 93%	Pseudoamino acid composition with physicochemical and statistical features
[108]	SVM	GPCR	Overall accuracy: 99.5%	Dipeptide composition of amino acids
[21]	SVM	GPCR	Overall accuracy: 89.8%–96.4%	Amino acid composition and dipeptide composition
[109]	SVM	GPCR	Overall accuracy: 99.6%	Pseudoamino acid composition
[110]	Adaboost	GPCR	Overall accuracy: 96.4% and MCC: 0.930	Pseudoamino acid composition with approximate entropy and hydrophobicity patterns
[111]	PCA	GPCR	Overall accuracy: 80.47–99.5%	Sequence derived features

**Table 7:** Summary of computational intelligence techniques in prediction of membrane protein.

Reference	CI techniques	Performance	Datasets
[112]	*k*-NN	Overall accuracy: 92.6%	PSSM and pseudoamino acid composition
[113]	*k*-NN	Overall accuracy: 87.65%	Protein sequence and PPI data
[114]	Fuzzy *k*-NN	Overall accuracy: 95.7%	Pseudoamino acid composition
[22]	OET-*k*-NN	Overall accuracy: 91.6%	Pseudoamino acid composition
[115]	SVM	Overall accuracy: 90.1%	Protein sequence
[116]	Discriminant analysis	Overall accuracy: 86.5%	Protein sequence information
[117]	Ensemble classifier	Overall accuracy: 91.2%	Pseudoamino acid composition and the approximate entropy

**Table 8:** Summary of computational intelligence for protein function prediction by using protein interaction network.

Reference	CI techniques	Performance	Datasets
[118]	Markov random field	Specificity: 45%, sensitivity: 64%	Functional probability of each protein
[119]	Network flow based algorithm	Accuracy: 10–90%	Structure of protein interactive maps
[120]	Neighbor based techniques	Precision: 0.9-1.0, recall: 98%	Label 1 and label 2 neighbors
[121]	Association analysis based method	Accuracy: 93%	H confidence, adjacency matrix
[122]	Naïve Bayes classifier	Precision: 49%, recall: 62%, MCC: 0.37	PPI data
[123]	RWR with *k*-NN	Accuracy: 58–73%	Neighborhood features
[124]	Time sequenced subnetwork	Significant module: 95.95%	Integrating the gene expression data and PPI data
[125]	Gibbs sampling based bootstrapping	TP/FP: 0.5 to 1.5	Interaction and annotation data
[126]	Network based approach	Precision: 54.83%, *F*-score: 43.74%	Function-function correlation
[127]	Neighborhood majority voting system	Precision: 67.3%, recall: 40.30%	Diffusion state distance (DSD)

**Table 9:** Summary of computational intelligence for protein function prediction by using gene expression data.

Reference	CI techniques	Performance	Datasets
[128]	Multilayer perceptron	TP rate: up to 79.6%, FP rate: up to 97%	DNA array expression data
[129]	MRF with Bayesian	Sensitivity: 87%	PPI, genetic interactions, highly correlated gene expression network, protein complex data, and structural properties
[130]	SVM	Accuracy: 89.44	Gene expression data
[131]	Genetic programming	Accuracy: 92.50–98.7%	Gene expression data
[132]	Majority voting genetic programming	Accuracy: 81.82%	Gene expression data
[133]	Genetic programming	Accuracy: 94.9–99.27%	Gene expression data
[134]	Genetic programming	Accuracy: 95.24–100%	Gene expression values and constant values
[135]	Fuzzy nearest cluster	Top N accuracy: 65.27%	Gene expression data
[136]	*k*-means	Accuracy: 0.16–0.24	PPI and gene expression data
[137]	Hypergraph	Accuracy: 97.95%	Gene expression data
[138]	Discriminative local subspaces with SVM	Average precision: 63% and *F* score: 0.44	Gene expression data

**Table 10:** Summary of computational intelligence techniques in pathway analysis from gene expression data.

Reference	CI techniques	Performance	Datasets
[139]	Gene set enrichment analysis	Sensitivity: 0.78, specificity: 0.98, AUC: 0.94	Gene expression data with significance analysis of microarray
[140]	Linear discriminant analysis	Error rate: 10–15%	Covariance matrix with group relationships among variables
[141]	Random forest	Error rate: 11–17%	Gene expression data
[142]	Naïve Bayes, decision tree based ensemble classifier	Accuracy: 91.2% and *F*-measure: 0.787	Gene expression data
[143]	SVM, Bayesian approach, C5.0, and random forest	Error rate: 7–15%	Gene expression data
[144]	Bayesian approach	AUC: 90.56%, Accuracy: 75.7%	Single-nucleotide polymorphisms

**Table 11:** The results analysis of different classifiers to predict protein functions.

Computational intelligence based techniques		DNA	RNA	Membrane	Enzyme	Nuclear receptor	G-protein coupled receptor	Overall
Random forest	ACC	78.6	64.7	89.2	81.6	97.4	94.6	86.7
	MCC	0.74	0.71	0.86	0.74	0.94	0.97	0.84

**Support vector machine**	**ACC**	**81.3**	**68.4**	**95**	**92**	**96.7**	**99.5**	**91.5**
	**MCC**	**0.86**	**0.76**	**0.91**	**0.83**	**0.96**	**0.98**	**0.90**

*k*-nearest neighbor	ACC	66.9	60.3	96.8	66.3	76.8	94.8	78.8
	MCC	0.74	0.51	0.68	0.70	0.85	0.97	0.76

Naïve Bayes	ACC	64.8	86	84.4	60.7	98.8	97.2	80.7
	MCC	0.61	0.64	0.81	0.65	0.87	0.98	0.77

SVM with AAC	ACC	72.4	73.5	87.7	89.3	99.8	70.8	84.1
	MCC	0.83	0.81	0.90	0.82	0.74	0.82	0.82

SVM with AAC + DC	ACC	82	71.3	95	91.8	96.9	94.3	91
	MCC	0.86	0.78	0.91	0.84	0.94	0.95	0.89
